# High-Resolution Structure of the N-Terminal Endonuclease Domain of the Lassa Virus L Polymerase in Complex with Magnesium Ions

**DOI:** 10.1371/journal.pone.0087577

**Published:** 2014-02-07

**Authors:** Gregor D. Wallat, Qinfeng Huang, Wenjian Wang, Haohao Dong, Hinh Ly, Yuying Liang, Changjiang Dong

**Affiliations:** 1 Biomedical Science Research Complex, School of Chemistry, University of St. Andrews, Fife, St. Andrews, United Kingdom; 2 Department of Veterinary and Biomedical Sciences, University of Minnesota, Twin Cities, Saint Paul, Minnesota, United States of America; 3 Laboratory Department of Surgery, The First Affiliated Hospital, Sun Yat-sen University, Guangzhou, Guangdong, China; 4 Norwich Medical School, University of East Anglia, Norwich Research Park, Norwich, United Kingdom; University of Edinburgh, United Kingdom

## Abstract

Lassa virus (LASV) causes deadly hemorrhagic fever disease for which there are no vaccines and limited treatments. LASV-encoded L polymerase is required for viral RNA replication and transcription. The functional domains of L–a large protein of 2218 amino acid residues–are largely undefined, except for the centrally located RNA-dependent RNA polymerase (RdRP) motif. Recent structural and functional analyses of the N-terminal region of the L protein from lymphocytic choriomeningitis virus (LCMV), which is in the same *Arenaviridae* family as LASV, have identified an endonuclease domain that presumably cleaves the cap structures of host mRNAs in order to initiate viral transcription. Here we present a high-resolution crystal structure of the N-terminal 173-aa region of the LASV L protein (LASV L173) in complex with magnesium ions at 1.72 Å. The structure is highly homologous to other known viral endonucleases of arena- (LCMV NL1), orthomyxo- (influenza virus PA), and bunyaviruses (La Crosse virus NL1). Although the catalytic residues (D89, E102 and K122) are highly conserved among the known viral endonucleases, LASV L endonuclease structure shows some notable differences. Our data collected from *in vitro* endonuclease assays and a reporter-based LASV minigenome transcriptional assay in mammalian cells confirm structural prediction of LASV L173 as an active endonuclease. The high-resolution structure of the LASV L endonuclease domain in complex with magnesium ions should aid the development of antivirals against lethal Lassa hemorrhagic fever.

## Introduction

Lassa fever virus (LASV) has natural hosts in rodents and can cause severe and lethal hemorrhagic fever diseases in humans. The virus is estimated to infect 300,000 to 500,000 people annually and is responsible for 5,000 deaths per year in many endemic areas of West Africa [1]. LASV is also a potential global heath threat as cases of infection have been reported in America, Europe and Asia [2], [3], [4], [5], [6]. Currently, no vaccine is available to prevent LASV infections. The only drug available to treat LASV infection is ribavirin, but to be effective, it has to be administered early when the disease is insidious and therefore it is difficult to distinguish LASV infection from other febrile diseases [1], [2].

LASV belongs to the *Arenaviridae* family, which consists of enveloped viruses with a bisegmented single-stranded RNA genome. Using an ambisense coding strategy, LASV genome encodes four proteins: glycoprotein complex (GPC), nucleoprotein (NP), matrix protein (Z), and the L RNA-dependent RNA polymerase. The GPC is proteolytically cleaved by the cellular signal peptidase and subtilase SKI-1/S1P into a stable signal peptide SSP and mature glycoproteins GP1 and GP2 [7]. The SSP/GP1/GP2 tripartite complex forms surface envelope spikes of the virus and is anchored on the viral membrane. The highly abundant NP encapsulates viral RNAs into ribonucleoprotein (RNP) complexes that also contain the L polymerase protein. The NP-bound viral RNAs serve as templates for both RNA replication and transcription [8], [9], which are facilitated by the L polymerase and NP proteins. In addition to its functions in viral RNP structural formation and genome transcription and replication, NP also strongly suppresses type I interferon (IFN) production via a unique immune evasion mechanism. We and others have recently shown that NP has 3′–5′ exoribonuclease activity with a preference for cleaving dsRNA substrates and that this viral exoribonuclease function is essential for mediating host immune suppression [10], [11], [12]. A small matrix protein (Z) mediates viral budding [13] and also regulates viral RNA replication and transcription [14], [15], [16].

Like orthomyxoviruses and bunyaviruses, arenaviruses cannot synthesize *de novo* the cap structure that is required to initiate viral mRNA synthesis. Instead, these viruses steal the caps from host mRNAs in a process termed cap snatching. Analysis of the non-templated RNA sequences in virally infected cells suggests that the snatched cap structures in arenaviruses contain 1 to 4 ribonucleotide(s), which are much shorter than those of influenza and bunyaviruses [17], [18], [19]. The cap snatching mechanism is most extensively studied in the influenza virus system. Influenza viral polymerase is a tripartite protein complex, which consists of the subunits PA, PB1, and PB2, each with specific functions in viral RNA replication and transcription. Structural analysis as well as biochemical and cell-based viral RNA synthesis assays demonstrate that PB2 binds the cap structure of host mRNAs [20] while the N-terminal domain of the PA subunit endonucleolytically cleaves the cap structure from these mRNAs [21], [22]. In arenaviruses, mutagenesis studies have revealed that the N-terminal domain of the LASV L protein, which consists of 250 amino acid residues, is uniquely required for viral transcription but not replication [23], implicating its potential role in cap snatching. Indeed, the crystal structure of the N-terminal region of the LCMV L protein (PDB: 3JSB) [24] reveals an endonuclease domain that is structurally homologous to the endonuclease domain of the influenza PA protein (PDB: 3EBJ) and that of the La Crosse (LACV) L polymerase (PDB: 2XI5) [25]. The endonuclease domain of PA was solved in complex with metal and monoribonucleotide and revealed the five potential catalytic residues H41, E80, D108, E119 and K137 [21], [22], [26], while LACV endonuclease domain was crystallized with metal and endonuclease inhibitor 2,4-dioxo-4-phenylbutanoic acid (DPBA) that were coordinated by five potential catalytic residues: H34, D52, D79, D92, and K94 [24]. The catalytic residues of the endonuclease domain of the arenavirus LCMV L polymerase were less clear. Although the L proteins of LCMV and other arenaviruses share some sequence conservation in the N-terminal endonuclease domain, sequence alignment fails to identify the conserved His of the expected H…PD…D/E…K motif. Crystal structure of the LCMV endonuclease domain suggests E51 (rather than His), D89, E102, D119, K115 and K122 as active site residues [25]. We believe that having additional structures of arenaviral L endonucleases in complex with metal and monoribonucleotide may help to clarify this ambiguity. Since endonuclease function is central in the viral cap snatching process, an impairment of this event can potentially block viral genome transcription and inhibit virus replication. Therefore, we believe that solving the structure of the endonuclease domain of the highly pathogenic LASV may help to pave the way for drug discovery to combat this deadly disease. Toward this end, we report here for the first time the crystal structure of the endonuclease domain of the LASV L protein in complex with Mg^2+^ ions at 1.72 Å and demonstrate its enzymatic activity required for viral RNA transcription. Our new structure sheds important lights on the molecular mechanism of cap snatching by arenaviruses and provides structural basis for the development of antiviral drugs targeting this essential step in the arenaviral life cycle.

## Materials and Methods

### Plasmids and DNA Cloning

DNA fragments encoding the N-terminal regions of different lengths (residues 1–190, 1–200, 1–250, 1–300 and 1–500) from the LASV L protein (L190, L200, L250, L300 and L500) were amplified by PCR with respective pairs of primers and cloned into the pMALC2X derivative plasmid pLou3 [10] and the pEHISTEV plasmid, which includes a hexahistidine tag and a tobacco etch virus protease cleavage site at the N-terminus of the cloned genes. Based on the structure, a new plasmid construct was designed to express the first 173 amino acids of the LASV L protein (L173) from the pEHISTEV plasmid. Three alanine substitution mutations (E51A, E102A and D119A) were generated in the backbone of L173 by site directed mutagenesis according to a protocol by Liu et al [27]. For protein expression in mammalian cells, the full-length LASV L fused with a myc tag at the C-terminus was cloned into the pCAGGS vector. Mutations were generated by PCR and cloned into the pCAGGS-L-myc vector. All constructs were confirmed by DNA sequencing. Primer sequences can be provided upon request.

### Protein Expression

The bacterial expression plasmids of LASV L N-terminal regions, with or without mutations, were individually transformed into Rosetta (DE3) cells. For L200, L250, L300, and L500, cells were propagated in 500 ml of LB medium containing appropriate antibiotics (25 µg/ml of kanamycin or 50 µg/ml of ampicillin and 34 µg/ml of chloramphenicol) in a 37°C shaker at 200 RPM overnight. The start-up culture was transferred to 10 L of Luria broth (LB) and grown at 37°C and 200 RPM until the optical density of the culture measured at a wavelength of 600 nm reached 0.5–0.7. At this stage, the expression of recombinant proteins was induced by the addition of IPTG to a final concentration of 0.2 mM and grown at 16°C for additional 16–20 hours. Cells were harvested by centrifugation at 5000 RPM and 4°C for 15 minutes. For L173 and L173 mutants, bacterial cells were grown in 400 ml auto-induction medium in 2 L flasks at 20°C and 250 RPM for 48 hours in the presence of kanamycin (25 µg/ml) and chloramphenicol (34 µg/ml).

### Protein Extraction and Purification

Cell pellets were resuspended at 4°C in sample buffer (20 mM Tris-HCl pH 7.4, 300 mM NaCl, 10% glycerol) supplemented with DNase, EDTA-free protease inhibitor cocktail (Roche, UK) and phenylmethylsulfonyl fluoride (PMSF, Sigma, UK), and lysed by two passes through a cell disruptor (Constant Systems Ltd., UK) at 30 Kpsi. Lysed cells were centrifuged at 18,000 RPM and 4°C for 45 minutes to remove cellular debris. The supernatant was applied twice to a pre-equilibrated nickel-nitrilotriacetic acid (Ni-NTA) agarose resin to purify the hexahistidine (His_6_)-tagged target proteins. After the resin was washed with 40 column volumes (CV) of wash buffer (20 mM Tris-HCl pH 7.4, 600 mM NaCl, 10% glycerol and 10 mM imidazole) to remove non-specific proteins, target proteins were eluted with 2 CV of elution buffer (20 mM Tris-HCl pH 7.4, 150 mM NaCl, 10% glycerol, 300 mM imidazole). Following the elution, the target protein was desalted (Hiprep 26/10 desalt column, GE-Healthcare, UK) into a low imidazole buffer (20 mM Tris-HCl pH 7.4, 150 mM NaCl, 10% glycerol, 10 mM imidazole) to prevent protein precipitation during proteolytic cleavage of the fusion partner by TEV protease. To remove the fusion tag, the protein sample was filtered through a 0.45 µm filter (Merck Millipore, UK) and re-applied to re-equilibrated nickel resin. The flow-through containing the target protein was concentrated to a volume of 7.5 ml and applied to an equilibrated gel filtration column (Superdex 200, GE-Healthcare). Protein fractions were collected and protein purity was determined on pre-cast SDS-PAGE (NuPAGE, Invitrogen, UK). Fractions of highest purity were combined and concentrated to 7 mg/ml.

### Protein Crystallization

Protein crystallization was performed using the sitting-drop vapour diffusion technique. The purified N-terminal 200 residues of LASV L (L200) did not form crystals. However, crystallization was initiated after proteolysis of LASV L190 with subtilisin A in a 700∶1 ratio for 60–90 minutes on ice. Upon proteolytic treatment, the protein crystallized rapidly in various conditions with best crystals found in 100 mM Tris-HCl pH 8–9.5, 250 mM MgSO_4_ and PEG 10,000. Larger crystals grew after three days in 2 µl of protein and 2 µl of precipitant drops with 100 µl reservoir solution.

### Data Collection

Protein crystals were cryoprotected with reservoir solution containing 20% glycerol and frozen in liquid nitrogen. Data was collected using synchrotron radiation with an oscillation angle of 0.5° and 360 recorded images at 1 second exposure for each at the Diamond Light Source, UK.

### Data Processing and Structural Determination

The X-ray diffraction data was indexed and integrated using iMosflm [28], and scaled using Scala [29] in ccp4 suite. The crystal structure of LASV L N-terminal region was determined by molecular replacement using Phaser [30] and the N-terminal domain of LCMV (PDB: 3JSB) as a search model. Model building was completed in Coot [31] and structure refinements were carried out using Phenix [32].

### *In Vitro* Endonuclease Assays

A single-stranded 16-nucleotide RNA fragment with a fluorescence 6-carboxyfluorescein label (FAM-label) at its 5′ end was synthesized and HPLC purified by Eurogentec (Eurogentec, Belgium), and further purified using a 20% polyacrylamide/8 M urea gel. The endonucleolytic activity of the wild-type L173 was determined using the FAM-RNA as substrate in a control experiment in which the RNA substrate in solution containing 20 mM Tris, pH 7.5, 0.3 M NaCl, 10% glycerol and RNase inhibitor (RNaseIN, Promega, UK) was incubated with purified LASV L173 at 37°C for 20 minutes in varying combinations of EDTA and divalent metal ions. Metal ion preference was tested by incubation of FAM-RNA substrate and LASV L173 with 0.5 mM of MgCl_2_, MnCl_2_, CaCl_2_ and ZnCl_2_ at 37°C for 20 minutes. Endonucleolytic cleavage of the alanine substitution mutants E51A, E102A and D119A was performed by incubation of FAM-RNA in a 1∶10 ratio with the individual mutated version of the protein in the presence of 2.5 mM MgCl_2_ and RNase inhibitor at 37°C. Samples were taken after 0, 25, 40 and 90 minutes incubation time and stopped by the addition of EDTA pH 8.0 (100 mM final concentration) and 100% formamide. Samples were heated to 95°C for 5 minutes prior to separation on a 20% PAGE/8 M urea gel. Vertical gel electrophoresis was carried out at 45°C and under protection from light for 150 minutes. Immediately after electrophoresis, the gel was scanned at an absorbance of 500 nm using a Typhoon scanner and the intensity of the bands was quantified by the ImageJ software [33].

### LASV Minigenome Transcription Assay

The LASV minigenome (MG) assay was conducted as described previously [10]. In brief, 293T cells were transfected with the LASV L and NP expression vectors together with the *in vitro*-transcribed LASV-based luciferase-encoding MG RNAs. A beta-gal expression vector was included in each transfection to normalize for cell transfection efficiency. Luciferase (LUC) activity was determined at 24 hours post-transfection, normalized by beta-gal activity, and shown as x-fold increase over a control sample that lacks the L expression plasmid. Each reaction was conducted in triplicates and in at least two independent experiments.

## Results

### Purification and Crystallization of the LASV L Endonuclease Domain

DNAs encoding the first 200, 250, 300 and 500 amino acids of the LASV L polymerase were first cloned into the pLou3 plasmids and expressed in *E. coli* Rosetta (DE3) cells as described in “Materials & Methods”. After multiple attempts using different induction conditions and media, the L200 containing the first 200 amino acid residues of the polymerase was the only construct that expressed well and was easy to purify. However, attempts to crystallize this protein failed. Similarly, L190 containing the first 190 amino acid residues of L was expressed and purified at a high level but again did not yield any crystals. A limited proteolysis screen revealed that, after subtilisin A treatment to cleave off some amino acids at the C-terminus of the L190 protein, crystals appeared in 100 mM Tris-HCl pH 9.0, 250 mM MgSO_4_ and 20% PEG 10,000 after 1 day of incubation. The final protein product for crystallization consists of residues 1–173 based on the structure determined as described below.

### Crystal Structure of the L endonuclease Domain

**Figure 1:**
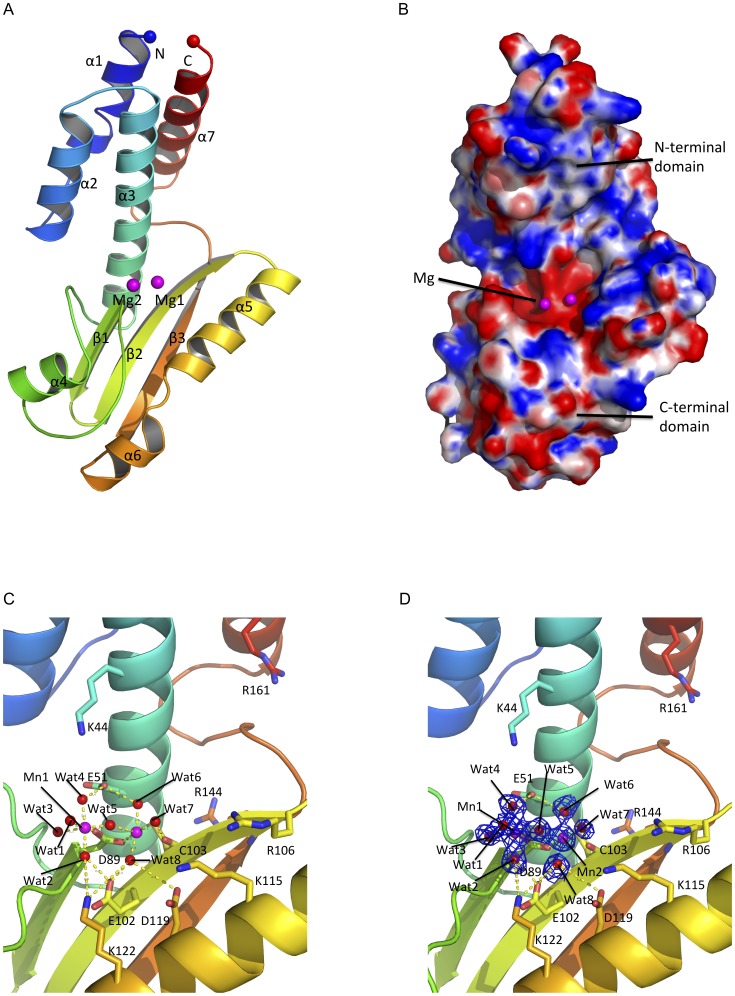
Structure of the LASV L endonuclease domain. **A,** The cartoon representation of the LASV endonuclease domain is shown in rainbow colors. The N-terminus is shown as a blue sphere and the C-terminus is shown as a red sphere. Magnesium ions are shown as magenta spheres. **B,** Electrostatic potential map of LASV L endonuclease domain. The highly positive charged residues are shown in blue (+10 K_b_T/e_c_) and highly negatively charged residues shown in red (−10K_b_T/e_c_). The catalytic cavity is located in vicinity to the magnesium ions. The putative RNA binding cleft is formed diagonally between the N-terminal and the C-terminal domains of the endonuclease. **C,** Atomic view of the active site of the LASV L N-terminal endonuclease domain. The two magnesium ions are coordinated by water molecules and the side chains of amino acid residue D89 and the main chains of C103. **D,** The original FoFc electron density map of the two magnesium ions and the water molecules contoured at 3σ.

The crystals of the LASV L N-terminal region belong to a space group of P4_3_2_1_2 with cell dimensions a = 57.72, b = 57.72, c = 134.51, and α = β = γ = 90°. The crystal structure was determined by molecular replacement using the LCMV endonuclease structure (3JSB) as the search model and refined to 1.72-Å resolution with an R_factor_ of 16.54% and the R_free_ of 17.83% (Table 1). The protein contains an N-terminal domain that consists of a four α-helices bundle (α1, α2, α3 and α7) and a C-terminal domain that composes of three antiparallel β-sheets (β1, β2 and β3) and two helices (α4 and α5) (Figure 1A). Between the two domains is a highly positively charged groove, which we speculate to be a RNA binding site, and a highly negatively charged cavity, which harbors two magnesium ions (Mg^2+^) and is a potential active site of the endonuclease domain (Figure 1B).

**Table 1:** Data collection and structure refinement statistics.

Data collection	LASV L endonuclease with two Mg^2+^ ions
Wavelength (Å)	0.9793
Space group	P4_3_2_1_2
Unit-cell parameters (Å; °)	57.72, 57.72, 134.51; 90, 90, 90
Resolution (Å)	40.81–1.72 (1.85–1.72)^a^
Unique reflections	24168 (2208)
Completeness (%)	96.65 (90.71)
Multiplicity	9.3 (9.7)
Mean I/σ (I)	43.65 (6.11)
R_merge_ (%)	0.048 (0.579)
**Refinement**	
R_factor_ b	16.51 (19.8)
R_free_ c	17.72 (25.43)
Number of atoms	1546
Protein	1386
Metal ions	2
Solvent	158
RMSD bond (Å)/angles (°)	0.010/1.12
Ramachandran favored (%)	99%
Mean B-factors (Å^2^)	22.9
Protein	21.9
Solvent	31.6
**PDB accession code**	4MIW

a Values in parentheses are for the highest resolution shell.

b R_factor_ = Σ||Fo|−|Fc||/Σ|Fo|, where Fo and Fc are observed and calculated as structure factors, respectively.

c R_free_ is calculated using 5% of total reflections, which is randomly selected as a free group and not used in refinement.

### The Potential Catalytic Residues of the Lassa L Endonuclease Domain

Two Mg^2+^ ions, presumably picked up by the protein from the crystallization solution, are located at the presumed active site of the LASV endonuclease domain. Four water molecules (Wat1, 2, 3 and 4) and the side chain of D89 coordinate the first Mg^2+^, while three water molecules (Wat4, 5 and 6), the side chain of D89 and the main chain oxygen of C103 coordinate the second Mg^2+^ (Figures 1C, 1D). This metal binding feature is different from that of the known structures of the influenza and LACV endonucleases, in which both metal ions are coordinated by four or three potential catalytic residues [21], [25] or by residue E80 and three water molecules [22]. Since endoribonuclease is a two-metal-dependent enzyme, potential catalytic residues must be located in close proximity to the two Mg^2+^ ions. In addition to D89, which coordinates the two Mg^2+^ ions, some other potential catalytic residues are E51 that coordinates Wat1 and Wat6, E102, which is 4.2 Å away from Wat4, D119, which is 5.2 Å from Wat5, K122, which is 5.7 Å from Wat3 and 5.8 Å from the first Mg^2+^, and H62, which interacts with E51 through side chains and is 6.2 Å away from Wat1. As the structure of the LASV L endonuclease domain does not contain an RNA substrate, we believe that, upon RNA binding, these potential catalytic residues may undergo significant conformational changes. Taken together, the structure of the LASV L endonuclease domain suggests that residues E51, D89, E102, D119, and K122 are potential catalytic residues, which is similar to those predicted for the LCMV endonuclease [24].

### Comparative Structural Analysis of the LASV L Endonuclease Domain with Other Known Viral Endonuclease Domains

**Figure 2:**
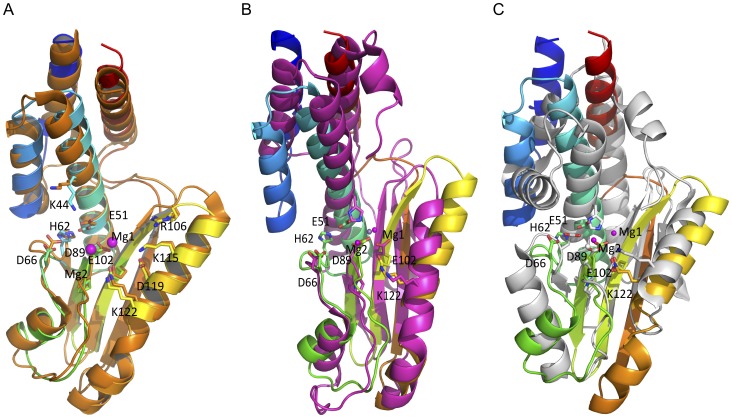
Similarities and differences between the structures of LASV L endonuclease domain and those of other negative-strand RNA viruses. Like in figure 1, the LASV L endonuclease domain is rainbow-colored. **A,** Structure of the LASV L endonuclease overlapping LCMV L endonuclease domain (orange). The residues are numbered based on the LASV L sequence. **B,** Superimposition of the structure of the LASV L endonuclease domain with the LACV L endonuclease domain (magenta). The magnesium ion of LACV is shown in gray, which is close to the position of the first magnesium ion in the LASV structure. **C,** Superimposition of LASV L endonuclease domain with influenza PA endonuclease domain (gray). The magnesium ion (gray sphere) from the structure of the influenza PA endonuclease is located close to the second magnesium ion of the LASV L endonuclease.

Three viral families, *Arena-, Bunya-*, and *Orthomyxoviridae* are known to use a similar cap-snatching mechanism to steal host mRNA cap structures for use in priming viral mRNA transcription. The available atomic structures of the viral endonuclease from each of these viral families, LCMV (*Arenaviridae*) [25], LACV (*Bunyaviridae*) [24], and influenza virus (*Orthomyxoviridae*) [21], [22] present an opportunity to compare the similarities and differences between these structures and that of the LASV L endonuclease reported in the current study (Figure S1). Structural comparison demonstrates that LASV L endonuclease closely resembles the L endonuclease of LCMV with rmsd of 1.382 Å over 166 Cα atoms [25]. Superimposition of the two structures shows identical positions of all the α-helices and β-sheets as well as at the potential catalytic residues E51, D89, E102, D119, and K122 (Figure 2A and Figure S2).

LASV L endonuclease also shows remarkable structural similarity to the LACV endonuclease structure with a rmsd of 3.018 Å over 128 Cα atoms (Figure 2B and Figure S2). In particular, the potential catalytic residues (E51, D89, E102, and K122), as well as residue P88 and a Mg^2+^ ion of LASV L endonuclease are located at similar positions as the corresponding active site residues (H34, D79, D92, and K108), as well as residue P78 and a Mg^2+^ ion of LACV NL1 [24]. Differences between the two structures are found at the N- and the C-terminal α-helices.

When LASV L endonuclease is compared to the PA endonuclease of influenza virus, their active sites show remarkable structural similarities with a rmsd of 3.703 Å over 103 Cα atoms, despite some obvious differences in both the N- and the C-terminal domains [21], [22] (Figure 2C and Figure S2). The potential catalytic residues D89, E102, K122 of LASV L endonuclease are located at the same positions as the catalytic residues D108, E119, K137 of influenza PA endonuclease. The E51 residue of LASV L is located at the same position as the H41 catalytic residue of influenza PA, suggesting that E51 may be a catalytic residue of the LASV L endonuclease. However, we notice that the active sites form a deep cavity in PA endonuclease but a flat surface in LASV endonuclease domain, which may partly explain our failure to obtain the LASV L in complex with single ribonucleotides despite multiple attempts.

Taken together, the LASV L endonuclease shows overall structural similarity to other viral endonucleases in the active sites, with some variations in the exact positions of several catalytic residues as well as in the N- and C-terminal regions. These structural features reflect a general mechanism of endonucleolytic cleavage, which is used by a diverse set of viruses. Nonetheless, these endonucleases each have their unique characteristics that may be involved in defining the substrate specificity and/or preference [21], [22], [24], [25].

### The N-terminal Domain of LASV L Exhibits Divalent Ion-dependent Endonuclease Activity *In vitro*

**Figure 3:**
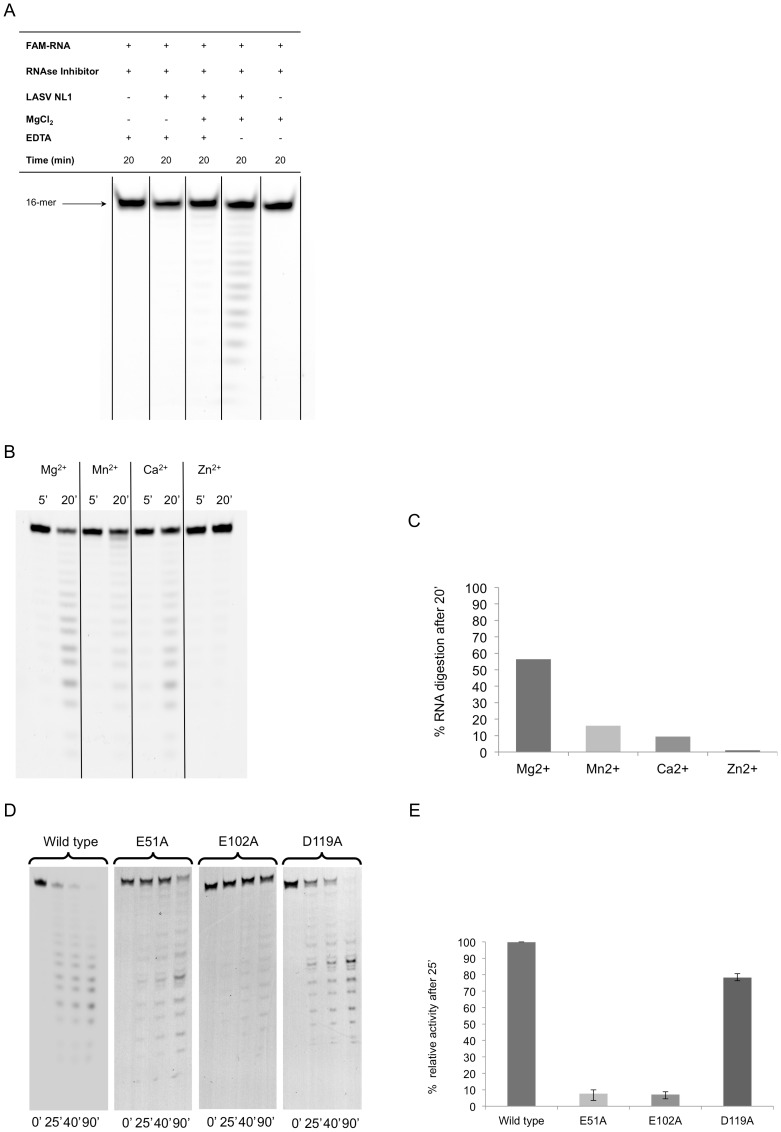
*In vitro* endonuclease activity of LASV L173. **A**, A 5′ FAM-labeled 16-nt single-stranded RNA substrate was incubated in a buffer with or without purified LASV endonuclease, with or without Mg^2+^ and with or without metal ion chelator EDTA, for 20 min at 37°C. The reaction products were separated by urea-PAGE and detected by fluorescence scanning. **B**, The *in vitro* endonuclease assay was conducted in buffers with different divalent cations for either 5 or 20 min. **C**, Percentage of RNA substrate degradation after 20 min incubation in a buffer with different divalent cations was quantified by fluorescence scanning. **D**, WT or mutant LASV L173 was analyzed by an *in vitro* endonuclease assay for 0, 25, 40 and 90 min. **E**, Percentage of RNA substrate degradation after 25 min was quantified by fluorescence scanning and normalized to WT control (set at 100%).

To verify its enzymatic activity, we first conducted an *in vitro* endonuclease assay, in which a 16-nt single-stranded RNA substrate is incubated for 20 minutes with purified LASV L173, MgCl_2_ and EDTA in varying combinations (Figure 3A). As MnCl_2_ at a concentration of 5 mM was found to cause precipitation of LASV L endonuclease, we used a final concentration of 0.5 mM of all divalent ions in the *in vitro* endonuclease assays throughout the study. LASV L endonuclease shows the highest levels of activity in the presence of Mg^2+^, followed by Mn^2+^ and Ca^2+^, and no activity in the presence of Zn^2+^ (Figures 3B, 3C). We then generated three LASV L endonuclease mutants that contain alanine substitution mutations at three of the potential catalytic residues (E51A, E102A, and D119A). When tested in the *in vitro* endonuclease assay, these mutant proteins exhibited different levels of defects in RNA cleavage. E51A and E102A showed significantly reduced endonuclease activities whereas D119A showed moderately impaired activity (Figures 3D, 3E).

### Mutational Analyses of the LASV L endonuclease Domain in Viral RNA Transcription

**Figure 4:**
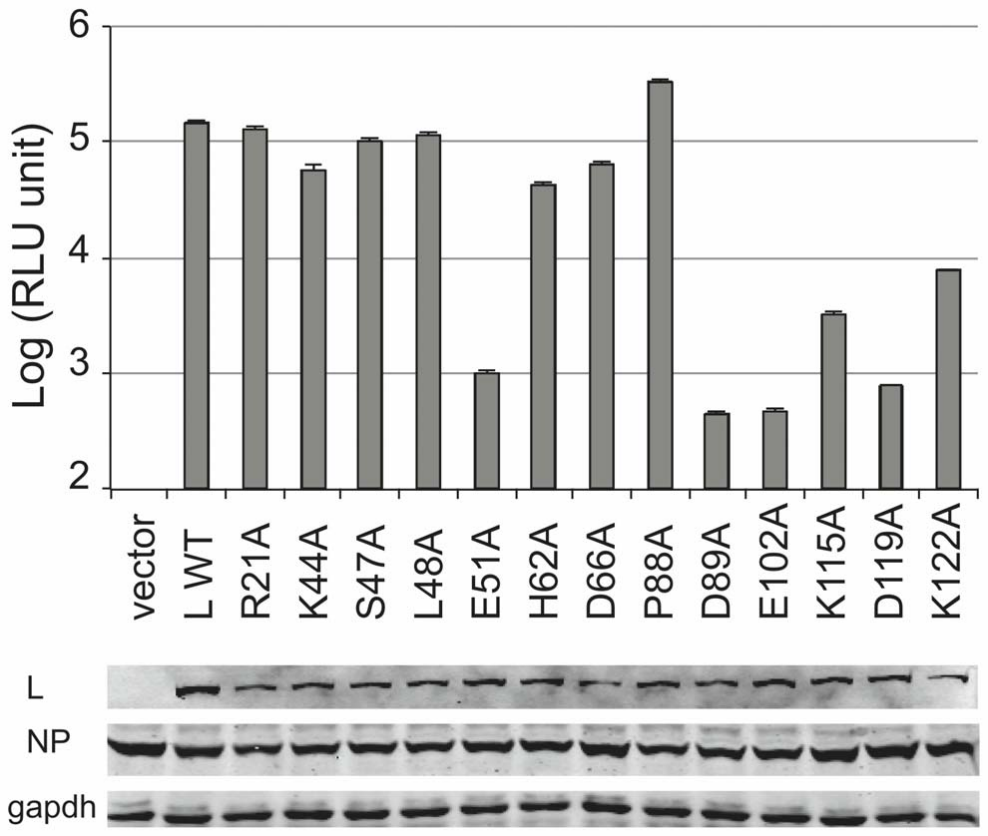
Mutational analysis of LASV L endonuclease domain in the LASV minigenomic RNA transcription assay. FLAG-tagged L expression vector with either WT or alanine substitution at the respective residue within the N-terminal endonuclease domain was used to transfect 293T cells, together with the myc-tagged NP expression vector and LUC-encoded LASV MG RNA. LUC activity was measured and plotted in log scale. Results shown are the average of at least three independent experiments with error bars representing standard deviations. The expression of L (WT or mutant), NP, and GAPDH in the transfected cells was detected by Western blot analysis using anti-FLAG, anti-myc, and anti-GAPDH antibody, respectively.

We next characterized the function role of potential catalytic residues within the LASV L endonuclease domain in mediating viral RNA transcription using the established mini-replicon (MG) assay as described previously [10]. Alanine substitution was introduced at each of the predicted catalytic residues of the endonuclease domain (E51A, D89A, E102A, D119A, and K122A) as well as at other residues found in close proximity to the catalytic residues (R21A, K44A, S47A, L48A, H62A, D66A, P88A, and K115A). These substitution mutations were made in a context of the myc-tagged full-length LASV L expression plasmid. 293T cells were transfected with wild-type (WT) or mutant L plasmid, nucleoprotein (NP) expression plasmid, and a Renilla luciferase LUC-encoding viral RNA molecule. Compared to WT, all L mutants appeared to be stably expressed, albeit at various reduced levels (Figure 4). As expected, catalytic mutants E51A, D89A, E102A, D119A and K122A as well as K115A found in close proximity to the other catalytic residues resulted in significantly reduced LUC expressions (by 2–3 logs). On the contrary, none of the other mutants R21A, K44A, S47A, L48A, H62A and D66A significantly reduced viral RNA transcription. Taken together, our LASV MG assay largely confirms previous mutational analysis of LASV and LCMV L proteins in viral RNA transcription [23], [25]. Published studies have demonstrated that targeted mutations at residues D89, E102, D119, K122, D129, E180, and R185 in the N-terminal region of LASV L protein significantly reduce the mRNA, but not complementary RNA levels, and that these residues might specifically be involved in the endonuclease process, which is required for viral RNA transcription but not replication. Remarkably, most of these residues overlap with the predicted catalytic residues of LASV L endonuclease domain as revealed by our crystal structure. Structural and functional analysis of LCMV L endonuclease also identified the conserved residue E51 as a potential catalytic residue [25]. Taken together, our structural and functional analyses of the LASV endonuclease domain coupled with previous analyses of both the LASV and LCMV L proteins have confirmed our structural prediction that residues E51, D89, E102, D119, and K122 are the catalytic residues of LASV L endonuclease.

## Discussion

Arenaviruses (e.g., LASV and LCMV), bunyaviruses and orthomyxoviruses steal the 5′ cap structures from host mRNAs to use as primers for viral RNA transcription. During this cap snatching process, a viral cap-binding protein recognizes the 5′ cap structure of host mRNA while a viral endonuclease cleaves the mRNA at some nucleotides downstream from the 5′ end. Recent studies have provided important insights into arenavirus proteins mediating the endonucleolytic cleavage. Previous mutational analyses of LASV L protein revealed the importance of its N-terminal region in viral RNA transcription [23] and sequence alignment shows its distant homology to the influenza virus PA endonuclease domain, implying that L contains an endonuclease domain at its N-terminus. The crucial evidence of L endonuclease activity was obtained from the biochemical and crystal structural characterizations of LCMV L N-terminal domain, which displays an *in vitro* endonuclease activity and exhibits overall homology to influenza PA endonuclease at a tertiary structural level. In this study, we report the first high-resolution crystal structure of LASV L endonuclease domain in complex with Mg^2+^. Our work confirms that arenavirus L polymerase proteins contain a conserved endonuclease domain at the N-terminal region. The structural information of LASV L endonuclease provides a vulnerable target for potential drug development to combat lethal Lassa fever and/or other pathogenic hemorrhagic fevers caused by arenaviruses.

Comparison of LASV L N-terminal domain to other endonucleases with known structures has shown various degrees of similarity in the overall protein folding. Not surprisingly, LASV L endonuclease shows the most structural homology to LCMV within the same *Arenaviridae* family, with identical positions for α-helices and β-sheets as well as the potential catalytic residues E51, D89, E102, D119, and K122, and the conserved H62 and K115 found in close proximity to the catalytic residues (Figure 2A, and Figure S2A). An interesting difference between LASV and LCMV L endonucleases is that the enzymatic activity of LCMV L is Mn^2+^ dependent and that it is more thermodynamically stable in the presence of Mn^2+^ ion [25], whereas LASV L precipitates in a solution that contains Mn^2+^ ion and is less active in Mn^2+^ than in Mg^2+^ (Figures 2A, 2B), the reason of which is unclear.

Based on structural and functional analyses of both LCMV and LASV L endonucleases, we believe that the potential catalytic residues of arenavirus endonuclease domain consist of E51, D89, E102, D119, and K122. Three of these catalytic residues D89, E102, and K122 have the same spatial location as those of LACV (Bunyaviridae) NL1 and influenza virus (Orthomyxoviridae) PA endonucleases (Figure S2, B and C). Apart from the three conserved catalytic residues common to all known viral endonucleases, LACV NL1 contains two other catalytic residues H34 and D52, while influenza virus PA contains H41 and E80. Interestingly, the conserved His catalytic residue, present in both LACV and influenza virus endonucleases, is uniquely absent in LCMV and LASV L endonucleases. Instead, both LCMV and LASV L encode a conserved E51 residue at the proximal position. The E51A mutation almost completely abolished viral mRNA transcription in the LASV MG assay (Figure 4) and has been shown to preferentially disrupt viral RNA transcription [24], suggesting that E51 is a potential catalytic residue of the arenaviral L endonucleases. Another residue located near the catalytic active site is D119 (Figure 1B), when mutated to alanine completely abolished viral RNA transcription (Figure 4) but not necessarily viral replication [23], strongly supporting its essential role in endonuclease catalytic activity. Taken together, the potential catalytic residues of arenaviral L endonucleases include E51, D89, E102, D119, and K122, which largely overlap with known viral endonucleases from other viral families but exhibit unique features of their own, suggesting similar but not necessary identical catalytic mechanisms.

**Figure 5:**
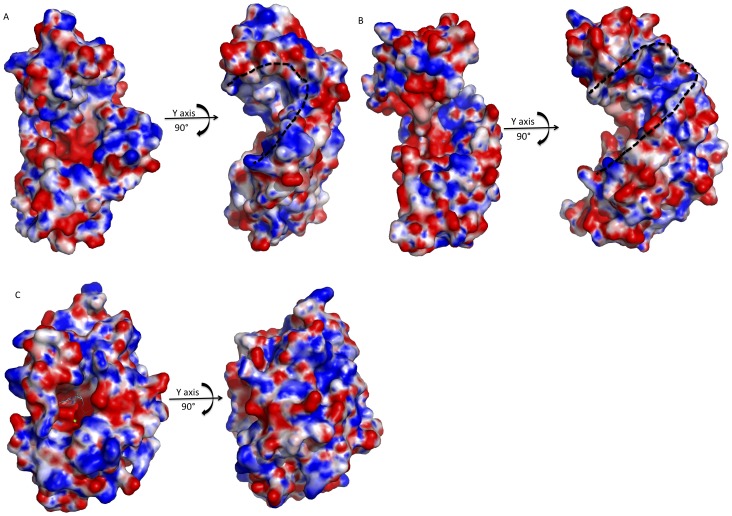
Electrostatic potential maps of the putative RNA binding cleft of known viral endonucleases. The black dotted lines outline the potential RNA binding clefts. **A**, The electrostatic potential maps of LCMV endonuclease. The putative RNA binding cleft were revealed by rotation by 90° along the y axis. **B**, The electrostatic potential map of LACV endonuclease. Rotation by 90° along the y axis shows the putative RNA binding cleft. **C,** The electrostatic potential maps of influenza PA endonuclease. The RNA binding cleft of the influenza endonuclease is closed/hidden. Rotation by 90° along the y-axis could not see the putative RNA binding cleft.

Crystal structural analysis of the LASV L N-terminal region also reveals a highly positively charged cleft consisting of residues K44, R106, K115, R144 and R161 located in between the N- and C-terminal domains (Figure 5A, dotted outline). These residues are conserved amongst known arenaviral L polymerases. Alanine substitutions of the respective residues abolished (or significantly reduced) both viral RNA transcription and replication, suggesting their essential roles in viral RNA synthesis. The functional mechanism of this positively charged cleft is unknown but it may potentially serve as the interface to bind RNA substrates for endonuclease and/or polymerase activities. Similar positively charged clefts can be identified in LACV L and influenza virus PA endonucleases, although their spatial locations do not overlap by superimposition [21], [22], [24]. Compared to arenavirus, LACV L endonuclease has a wider (or deeper) cleft [24] (Figure 5B), while influenza virus PA contains a blocked cleft, which likely requires conformational changes for substrate binding (Figure 5C) [21], [22]. Presumably this positively charged cleft of the endonucleases of the LASV, LACV and influenza virus PA plays an essential role in viral RNA synthesis. However, its exact functional role and mechanism in viral RNA transcription and replication need to be investigated further.

## Conclusions

We have provided the first high-resolution crystal structure of the LASV L N-terminal endonuclease domain in complex with magnesium ions and demonstrated that viral endonucleases from three separate viral families: *Arena-*, *Bunya-*, and *Orthomyxoviridae*, share some common features with some unique structural variations, suggesting that, although different viral families utilize a general mechanism to conduct the endonucleolytic cleavage of host mRNAs at their 5′ ends, each does it with some distinct characteristics. Further characterization of the common and unique features of these viral endonucleases may help develop specific antiviral therapeutics against these important human pathogens.

## Supporting Information

Figure S1 **Sequence alignment of the L N-terminal endonuclease domain of segmented negative-stranded RNA viruses of three families.** LASV, LCMV, LACV and H5N1 represent Lassa fever virus (josiah strain), Lymphocytic choriomeningitis virus (Armstrong strain), La Cross virus (mosquito/1978), and influenza virus (H5N1 strain).
(DOC)

Figure S2 **Superimposition of the active site of the LASV endonuclease structure with those of other viruses.** The balls in magentas are to signify Mg^2+^, while balls in grey are Mn^2+^. The residues are numbered according to the LAVS endonuclease. The LASV, LCMV, LACV and influenza endonucleases are shown in rainbow, orange, magentas and grey, respectively. A, Superimposition of the active site of the LASV endonuclease with that of the LCMV endonuclease, showing the complete conservation of the putative catalytic residues between the proteins. B, Superimposition of the active site of the LASV endonuclease with that of the LACV. C, Superimposition of the active site of the LASV endonuclease with that of the influenza virus.
(DOC)
