# Supplementary material for: High-Resolution Structure of the N-Terminal Endonuclease Domain of the Lassa Virus L Polymerase in Complex with Magnesium Ions
Source: PLoS One. 2014 Feb 7;9(2):e87577. doi: 10.1371/journal.pone.0087577 (PMC3917842; doi:10.1371/journal.pone.0087577)
Supplement: Figure S1 — Sequence alignment of the L N-terminal endonuclease domain of segmented negative-stranded RNA viruses of three families. LASV, LCMV, LACV and H5N1 represent Lassa fever virus (josiah strain), Lymphocytic choriomeningitis virus (Armstrong strain), La Cross virus (mosquito/1978), and influenza virus (H5N1 strain). (DOC) [file pone.0087577.s001.doc]

**Figure S1.** Sequence alignment of the L N-terminal endonuclease domain of segmented negative-stranded RNA viruses of three families. LASV, LCMV, LACV and H5N1 represent Lassa fever virus (josiah strain), Lymphocytic choriomeningitis virus (Armstrong strain), La Cross virus (mosquito/1978), and influenza virus (H5N1 strain).
