# Supplementary material for: High-Resolution Structure of the N-Terminal Endonuclease Domain of the Lassa Virus L Polymerase in Complex with Magnesium Ions
Source: PLoS One. 2014 Feb 7;9(2):e87577. doi: 10.1371/journal.pone.0087577 (PMC3917842; doi:10.1371/journal.pone.0087577)
Supplement: Figure S2 — Superimposition of the active site of the LASV endonuclease structure with those of other viruses. The balls in magentas are to signify Mg2+, while balls in grey are Mn2+. The residues are numbered according to the LAVS endonuclease. The LASV, LCMV, LACV and influenza endonucleases are shown in rainbow, orange, magentas and grey, respectively. A, Superimposition of the active site of the LASV endonuclease with that of the LCMV endonuclease, showing the complete conservation of the putative catalytic residues between the proteins. B, Superimposition of the active site of the LASV endonuclease with that of the LACV. C, Superimposition of the active site of the LASV endonuclease with that of the influenza virus. (DOC) [file pone.0087577.s002.doc]

A B

C

**Figure S2.** Superimposition of the active site of the LASV endonuclease structure with those of other viruses. The balls in magentas are to signify Mg^2+^, while balls in grey are Mn^2+^. The residues are numbered according to the LAVS endonuclease. The LASV, LCMV, LACV and influenza endonucleases are shown in rainbow, orange, magentas and grey, respectively. A, Superimposition of the active site of the LASV endonuclease with that of the LCMV endonuclease, showing the complete conservation of the putative catalytic residues between the proteins. B, Superimposition of the active site of the LASV endonuclease with that of the LACV. C, Superimposition of the active site of the LASV endonuclease with that of the influenza virus.
